# Turning to Religion During COVID-19 (Part I): A Systematic Review, Meta-analysis and Meta-regression of Studies on the Relationship Between Religious Coping and Mental Health Throughout COVID-19

**DOI:** 10.1007/s10943-022-01703-5

**Published:** 2023-01-02

**Authors:** Daniel Pankowski, Kinga Wytrychiewicz-Pankowska

**Affiliations:** 1 Faculty of Psychology, University of Warsaw, Stawki 5/7, 00-183 Warsaw, Poland; 2 University of Economics and Human Sciences in Warsaw, Warsaw, Poland

**Keywords:** Religious coping, COVID-19, Quality of life, Wellbeing, Post-traumatic growth

## Abstract

The COVID-19 pandemic and the many associated socio-economic changes constitute a stressful event that required adaptation to new, dynamic, and often threatening conditions. According to the literature, coping strategies are one of the factors that determine a person’s degree of adaptation to stressful situations. A systematic review and meta-analysis was performed on the relationship between religious coping and selected indicators of mental health. Due to the large amount of data, this work has been divided into two parts: this first part discusses positive mental health indicators, while the second discusses negative mental health indicators (Pankowski & Wytrychiewicz-Pankowska, 2023). A systematic review of PubMed, Science Direct, the Cochrane Library, Google Scholar, the Database of Abstracts of Reviews of Effects, and Google Scholar databases was carried out. In addition to the synthesis of information obtained from the research, a meta-analysis of correlation was also performed to determine the strengths of the relationships between the analysed variables, and selected moderators were assessed using meta-regression. Quality of life, well-being, satisfaction with life, happiness, and post-traumatic growth were the positive mental health indicators considered. Meta-analyses indicated a statistically significant relationship between positive religious coping and flourishing (well-being) with overall correlation values of 0.35 [0.30; 0.40]. Further calculations also indicated a relationship between negative religious coping and flourishing − 0.25 [− 0.34; − 0.15]. Data synthesis shows associations between religious coping and such indicators as satisfaction with life and post-traumatic growth, but these issues require further investigation.

## Introduction

The COVID-19 pandemic was an event that had a massive effect on the world around us. It affected practically all spheres of life: physical health (Daher et al., 2020), the economy (Ashraf, 2020), education (Marinoni et al., 2020), work (Kramer et al., 2020), mobility (Abu-Rayash et al., 2020), and mental health (MH; Bourmistrova et al., 2022; Samji et al., 2022). Isolation from loved ones (both voluntary for fear of their health and due to quarantine), rising prices and/or scarcity of consumer goods, paralysis of health care systems, economic uncertainty, including layoffs and bankruptcy, remote work/education without physical contact with other people, and many other factors contributed to reduced quality of life and greater levels of depressive symptoms and anxiety (Brenner et al., 2020; Rumas et al., 2021). The reality created by the pandemic made it necessary for many people to reorganize their day-to-day lives (Cancello et al., 2020). It should be noted that the COVID-19 situation was a new one for the vast majority of people—recent pandemics did not reach such a scale and did not last as long. In addition to its indirect impact (changes and limitations resulting from the pandemic), COVID-19 also directly influenced the functioning of people who became infected. In addition to long COVID (Crook et al., 2021), serious health problems (e.g. ECMO treatment or other systemic complications, see: Gribensk et al., 2022; Shanbehzadeh et al., 2021) could lead to the emergence and worsening of mental health difficulties.

There is no doubt that the pandemic was a major stressor that, to some extent, affected the vast majority of people in the world. According to the assumptions of the theoretical models used in research on stress, strategies for coping with stress are one of the factors that regulates the consequences of a given stressful situation. Lazarus and Folkman (1984) described coping as different types of deliberate efforts (cognitive, emotional, and behavioural) aimed at resolving a given stressful situation through either addressing the problem itself or the resultant emotions (including avoidance). In a situation that is assessed as stressful (cognitive appraisal process), actions are taken to restore the previous balance. An individual may use a variety of coping strategies depending on, among other things, their availability or subjective assessment of their effectiveness in a given situation or based on previous experience (Lazarus et al., 1984). One strategy used in stressful situations is religious coping (RC), which involves the use of various types of religious practices. Religious coping can, for example, help one to reinterpret a stressor (e.g. “it is God's will”), or provide emotional support, both from God and from religious communities. RC can be divided into positive religious coping (pRC) and negative religious coping (nRC). The former refers primarily to a sense of connectedness with a transcendent force and a secure relationship with a caring God, while the latter refers to feeling abandoned or punished by God as well as interpersonal religious discontent (Pargament et al., 2011).

The coping process ends when a given situation no longer causes stress (which does not necessarily mean that the stressor has been eliminated), which is largely dependent on subjective appraisal; a given stressor may also be successfully ignored, so that it no longer functions as a source of stress (Lazarus et al., 1984). The effects of the coping process can be operationalized in many ways, but most often the effectiveness of a given strategy is determined by its impact on MH. Commonly used indicators include, for example, quality of life, level of anxiety, or the severity of depressive symptoms (De Ridder et al., 2008). The issue of the negative impact of the pandemic on mental health has been the subject of much analysis during the pandemic. Numerous studies have indicated, inter alia, high prevalences of depression, anxiety (Salari et al., 2020), PTSD (Cénat et al., 2021), sleep problems (Jahrami et al., 2021), and other mental health issues. However, in addition to negative mental health indicators, many studies have also focused on positive aspects, such as life satisfaction and flourishing. An important issue here is the fact that a high intensity of negative MH indicators (such as anxiety or depression) does not necessarily mean a low level of positive indicators, such as life satisfaction or happiness (see: Seligman, 2008); therefore, these two issues should be analysed separately, taking into account the broadest possible repertoire of variables that may be affected by stressful situations. These studies sought to identify the factors responsible for high levels of negative indicators, as well as protective factors that could buffer the negative effects of the pandemic.

Therefore, we decided to conduct a systematic review of the literature on the relationship between religious coping and mental health indicators, in particular, focusing on both negative determinants of mental health, such as stress, depression, and anxiety, as well as positive determinants, such as post-traumatic growth, well-being (WB), and quality of life (QoL). Due to, inter alia, the fact that studies were conducted all over the world, the observed relationships between RC and MH indicators differed between studies. Therefore, we attempted to identify factors that may play significant roles in determining the strength of these relationships by performing a meta-regression of relevant studies.

## Methods

### Search Strategy

This review was pre-registered at OSF (Open Science Framework) below the link: osf.io/54ygr (https://doi.org/10.17605/OSF.IO/GMNFV) and after the registration DP and KWP independently conducted systematic literature searches using the PRISMA protocol (Moher et al., 2009). The reviewers searched PubMed, Science Direct, the Cochrane Library, Google Scholar, and the Database of Abstracts of Reviews of Effects using the keywords: "religious coping OR religion coping OR spiritual coping AND COVID". In the review, it was decided to also include articles from the "grey zone" (Google Scholar) only when both authors, having carefully read the text, had no doubts about the methodological soundness and quality of the article (see Appendix 1). Each article was independently assessed by both authors.

The search was limited to the English language, but no limitation was placed on the ethnicity of the human participants. The reference lists of relevant articles were also reviewed for other relevant articles.

### Selection Criteria

**Fig. 1:**
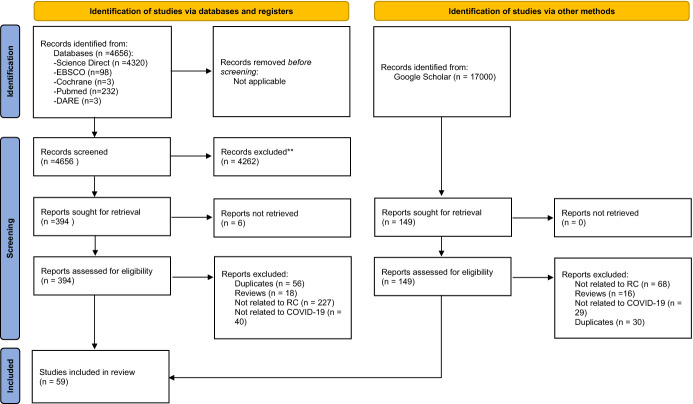
Flow diagram of studies identified, excluded and included in the systematic review

Based on the presence of the searched-for or synonymous terms, 394 articles were selected for further analysis. The process for selecting articles is shown in Fig. 1. Ultimately, the following criteria were adopted for the systematic review: (1) the study must refer to RC; (2) the study must describe a relationship between RC and mental health; (3) the methods used in the study must have a described reliability; (4) it is clearly stated in the title or abstract that the research relates to the pandemic (or resultant difficulties, such as lockdown or distance learning). In our work, we focused on the mental health indicators operationalized as (a) presence/intensity of psychopathological symptoms such as PTSD, anxiety or depressive symptoms; (b) the level of functioning of a given person in various areas of life—defined as quality of life, and (c) positive indicators of adaptation, such as well-being, satisfaction with life or post-traumatic growth. Pre-prints, conference reports, and dissertations were not included in the review. We assumed that due to methodological issues, in the case of cross-sectional and longitudinal studies, the tools used to evaluate RC should have proven reliability. The review included only articles in which the instructions in the tool related to coping with stress—studies on, for example, the level of religiosity or the frequency of religious practices were excluded.

Furthermore, for the meta-analyses and meta-regressions, additional criteria were adopted: the analysis must have examined at least 3 studies that used the same method of assessing RC and MH and the articles must give the value of the correlation coefficient.

Two researchers (DP and KWP) independently reviewed 394 texts, identifying those that should be included in the analyses based on these criteria. In cases of disagreement, consensus was reached after discussion between the 2 reviewers. The reference lists were reviewed to identify other studies related to the topic.

### Data Extraction

DP and KWP independently extracted the data for each study. The variables of interest for the systematic review were: year of publication, country of origin, sample size, basic sociodemographic data (sex, age, population), methods of assessing RC and MH, and the main findings of the study.

For the meta-analysis, the strengths of the relationships between the two variables were extracted. Only the correlation coefficients were taken into account—when the relationships were reported using the beta value, the results were not included in the meta-analysis.

As potential moderators, we extracted: the percentage of women in the sample, the mean age in the study group, the proportion of people professing a given religion, the percentage of people declaring themselves to be believers, the groups examined (e.g. the general population or medics), the percentage of people who were infected with COVID-19 at the time of the study, the percentage of people who had been infected with COVID-19, the percentage of people in quarantine, the percentage of people who reported somatic symptoms (e.g. fever), the percentage of people with higher education, the percentage of people in a stable relationship (married), the percentage of people living in rural areas, the percentage of people living alone, and the percentage of people with chronic diseases. Due to differences in the introduction and nature of restrictions in different countries (e.g. local lockdowns), we decided to not examine as potential moderators the time when the research was conducted and the nature of the restrictions in each country.

The method for recording the data was agreed upon before coding the results. Inter-rater reliability was satisfactory; in cases of disagreement, consensus was reached after a discussion between the two reviewers.

### Quality Assessment

To evaluate the quality of the selected studies, we used an adapted version of the Newcastle–Ottawa cohort scale for cross-sectional studies (Modesti et al., 2016), which takes into consideration the selection of samples, comparability of subgroups, and exposure. Using this scale, we scored each study independently. Inter-rater compatibility was satisfactory, and scoring differences were reconciled through discussion (Appendix 1).

### Statistical Analysis

Meta-analysis and meta-regression were conducted in the R Studio platform (R Studio, Boston, MA, USA) using the R software environment; the “metacor” library was used to calculate correlations (Schwarzer, 2007). The meta-analysis used the inverse variance method with restricted maximum-likelihood estimator for tau^2^, *Q*-Profile method for confidence interval of tau^2^ and tau. Fisher's *Z* transformation of correlations was used. Overall rates and 95% confidence intervals were calculated using a random effect model. Funnel plots were used to determine the possibility of publication bias (Appendixes 2). The heterogeneity of effects across studies was estimated using the *Q* test and quantified using the *I*^*2*^ statistic. Meta-analyses were performed with random effects models. Any *p* values less than 0.05 were considered significant.

## Results

### Results of the Systematic Review

**Fig. 2:**
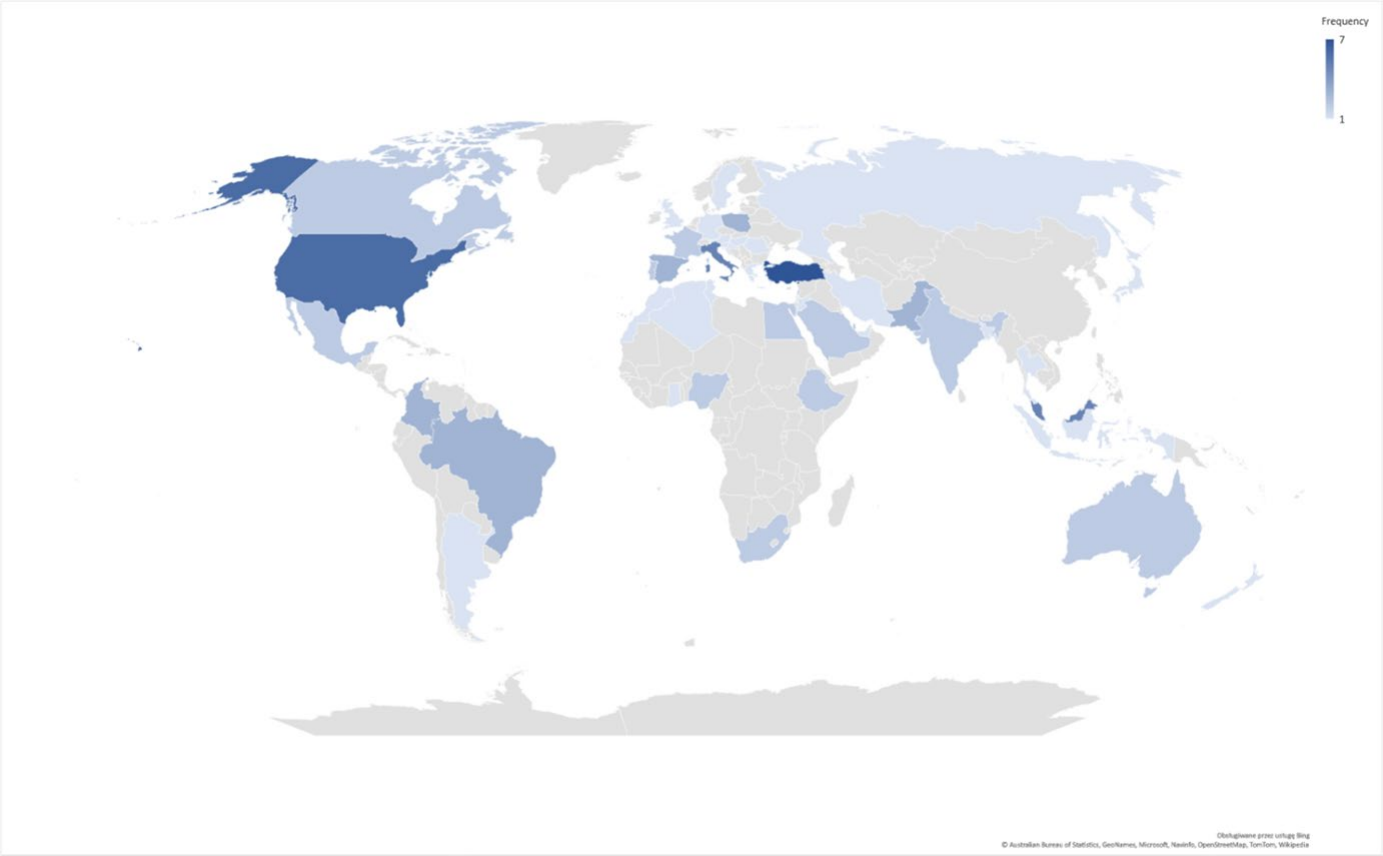
Choropleth map of numbers of studies by country

**Fig. 3:**
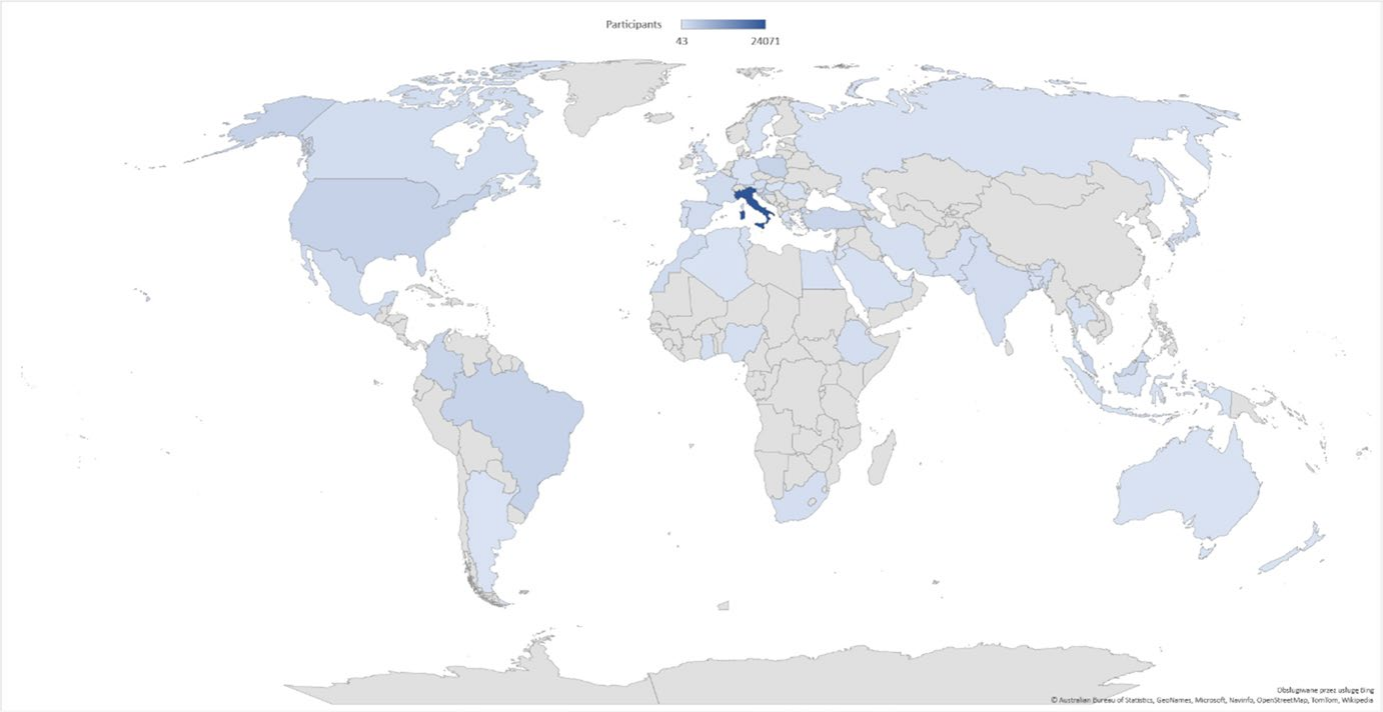
Choropleth map of participants by country

The search using the methodology described above yielded 59 studies that fulfilled all selection criteria for the systematic review. Altogether, 68,824 participants participated in these studies. The mean age of the participants ranged from 20.8 (Masha'al et al., 2022) to 76.3 (Willey et al., 2022) years. For details on the number of surveys and the number of respondents from each country, see Figs. 2 and 3, respectively, and Appendix 3.

For the sake of the readability of the results, we decided to analyse each aspect of mental health separately. It should be noted that a large part of the research focused on several MH indicators, so the numbers of studies and participants do not add up to the numbers given previously in the text. In the following subsections, selected indicators of MH are analysed in detail.

Due to the large amount of collected data, we decided to divide the article into two parts. In the first, positive MH indicators were analysed: quality of life, well-being, satisfaction with life (SWL), levels of happiness, and post-traumatic growth (PTG).

The second part of the review focuses on negative MH indicators: severity of depressive symptoms, anxiety, stress levels, symptoms of peri- and post-traumatic stress, and general negative MH indicators.

### Quality of Life

The systematic literature review identified 5 studies in which 3363 participants took part. The research was conducted from April 2020 to March 2021. A large variety of tools were used to assess RC, making it impossible to carry out a meta-analysis of the collected results. QoL was examined in over half of the studies using the WHO Quality of Life scale (WHOQOL-BREF). The exact results are shown in Table 1.

**Table 1:** Relationship between religious coping and quality of life

Authors [Country]	Sample *N* [group]	Date started*	Date finished*	Basic sociodemographic characteristics	Tools used	Main findings
Albani et al. (2022) [Greece]	200 [nursing students]	1 March, 2021	30 March, 2021	86.5% of the sample were female; mean age was 22.8 (*SD* = 12.2)	RC: Brief-RCOPE; QoL: SF-36	nRC was negatively associated with Role/Physical Functioning, Bodily Pain, General Health, Vitality, Social Functioning, Mental Health, Physical Component Summary, Mental Component Summary
Altunan et al. (2021) [Turkey]	205 [people suffering from multiple sclerosis]	n.i	n.i	74.1% of the patients were female; mean age was 37.7 (*SD* = 10.0)	RC: Brief-COPE; QoL: SF-12	RC was not correlated with QoL
Budimir et al. (2021) [Austria]	1005 [GPs]	10 April, 2020	30 April, 2020	52.7% of the sample were female; n.i. about mean age	RC: SCI; QoL: WHOQOL BREF	RC was positively associated with QoL
Shamblaw et al. (2021) [Canada]	T1: 797; T2 395 [GPs]	21 April, 2020	27 May, 2020	T1: 54.6% of the sample were female; mean age was 32.2 years (*SD* = 11.5); T2: 55.7% of the sample were female; The mean age was 33.7 years (*SD* = 12.6)	RC: Brief-COPE; QoL: WHOQOL BREF	RC was positively associated with QoL at T1
Vitorino et al. (2021) [Brazil]	1156 [GPs]	11 May, 2020	3 June, 2020	69.6% of the sample were female; mean age was 37.6 years (*SD* = 14.0)	RC: SRCOPE-14; QoL: WHOQOL-BREF	nRC was negatively associated with Physical, Psychological, Social Relationships, and Environment QoL. pRC was positively associated with Psychological, Social Relationships, and negatively with Environment QoL

*RC* Religious coping, *Brief-RCOPE Brief* Religious COPE, *QoL* Quality of Life, *SF-36* 36 item Short Form Health Survey, *nRC* negative religious coping, *n.i.* no information, *Brief COPE* Brief Coping Orientation to Problems Experienced, *GP* general population, *SCI* Stress and Coping Inventory, *WHOQOL-BREF WHO* Quality of Life-BREF, *T1* measurement 1, *T2* measurement 2, *SRCOPE-14* Brief Scale for Spiritual/Religious Coping, *pRC* positive religious coping

*In studies that did not specify the exact dates on which data were collected (months only), the beginning (1) and end (30) of the month were used as the starting and ending points. nRC—negative religious coping; pRC—positive religious coping

### Well-being

The systematic literature review identified 10 studies addressing the relationship between RC and WB, with a total of 17,469 participants. The research was conducted from February 2020 to December 2021. The Brief-COPE was used to evaluate RC in half of the studies (*n* = 5), while WB was assessed using various tools, including the PERMA Profiler (*n* = 4) and Flourishing Scale (*n* = 3). Most studies (except two) showed a relationship between RC and WB. The exact results are shown in Table 2.

**Table 2:** Religious coping and psychological well-being

Authors [Country]	Sample *N* [group]	Date started*	Date finished*	Basic sociodemographic characteristics	Tools used	Main findings
Budimir et al. (2021) [Austria]	1005 [GPs]	10 April, 2020	30 April, 2020	52.7% of the sample were female; n.i. about mean age	RC: SCI; WB: WHO-5	WB was positively associated with RC
Counted et al. (2022) [Colombia and South Africa]	1172 [Study 1: Colombian students] and 451 [Study 2: South Africans]	3 April, 2020	25 May, 2020	Women were 62.12% of the sample in Study 1 and 65.85% in Study 2. Mean age was 21.70 (*SD* = 3.96) in Study 1 and 33.54 (*SD* = 11.93) in Study 2	RC: RCOPE; FI	A positive association found between p RC and WB. There was a medium-sized negative correlation between nRC and WB
Davis et al. (2021) [USA]	T1 (1 month prepandemic): 1036; T2 (1 month into the pandemic): 453; T3 (3 months into the pandemic): 302 [GPs]	6 February, 2020	6 June, 2020	Women were 47.4% of the sample; n.i. about age	RC: Brief-RCOPE; WB: FS	No relationship between RC and WB
Eisenbeck et al. (2022) [Algeria Argentina Australia Bangladesh Brazil Canada Colombia Egypt France Germany Hungary India Indonesia Italy Lebanon Mexico New Zealand Nigeria Pakistan Poland Portugal Romania Russia Slovenia Spain Sweden Thailand Turkey UK USA]	11,227 [GPs]	1 March, 2020	30 June, 2020	69.9% of the sample were women; mean age was 35.36 (*SD* = 13.26)	RC: Brief-COPE; WB: PERMA	No relationship between RC and WB
Gupta et al. (2022) [whole world]	369 [hospitality and tourism lecturers]	1 February, 2021	30 April, 2021	53.66% of the sample were female; age 18–25: 16.53%; 26–35: 23.31%; 36–45: 27.9%; 46 + : 32.25%	RC: Brief-COPE; WB: WHO Index of Well-being; PERMA-Profiler	RC was positively correlated with the WHO Index of Well-being
Habib et al. (2020) [Pakistan]	200 [GPs]	n.i	n.i	n.i. about distribution of sex in the sample; n.i. about mean age of participants	RC: RCOPE; WB: BBC subjective well-being scale	pRC was a significant predictor of positive affect and life satisfaction, while nRC was significant predictor of negative affect
Lopes and Nihei (2021) [Brazil]	1224 [undergraduate students]	14 September, 2020	19 October, 2020	68.6% of the sample were female; age: 18–24: 77.9%; > 24: 22.1%	RC: Brief-COPE; WB: Psychological Well-Being (PWB)	RC was positively associated with environmental mastery, personal growth, positive relations with others, purpose in life, and self-acceptance
MacIntyre et al. (2020) [International]	634 [language teachers]	5 April, 2020	19 April, 2020	80% of the sample were female; age: < 22:25.4%; 22–32: 19%; 33–43: 31%; 44–54: 32%; 55–65: 13%; > 65: 1%	RC: Brief-COPE; WB: WHO Index of Well-being; PERMA-Profiler	RC was positively correlated with the WHO Index of Well-being
Moussa et al. (2022) [Lebanon]	333 [students]	1 November, 2021	30 December, 2021	65.8% of the sample were female; mean age was 22.95 (*SD* = 4.79)	RC: Brief-RCOPE; WB: FS, Positivity Scale	pRC was positively associated with positivity and flourishing; nRC was negatively related with positivity and flourishing
Umucu and Lee (2020) [USA]	269 [self-reported disabilities and chronic conditions]	1 April, 2020	30 April, 2020	43.9% of the sample were female; The mean age was 39.37 years (*SD* = 12.18)	RC: Brief-COPE; WB: PERMAProfiler,	RC was positively associated with WB

*GP* general population, *RC* religious coping, *W-B* well-being, *WHO-5* World Health Organization—Five Well-Being Index, *RCOPE* Religious COPE, *FI* Flourishing Index, *FS* Flourishing Scale, *pRC* positive religious coping, *nRC* negative religious coping, *T1* measurement 1, *T2* measurement 2, *T3* measurement 3, *PERMA* positive emotion, engagement, relationships, meaning, and accomplishment, *n.i.* no information, *PWB* psychological well-being

*In studies that did not specify the exact dates on which data were collected (months only), the beginning (1) and end (30) of the month were used as the starting and ending points

#### Meta-analysis

The analysis of the studies included in the review only allowed for a meta-analysis of the relationship between flourishing and RC assessed with the Brief-RCOPE.

**Fig. 4:**
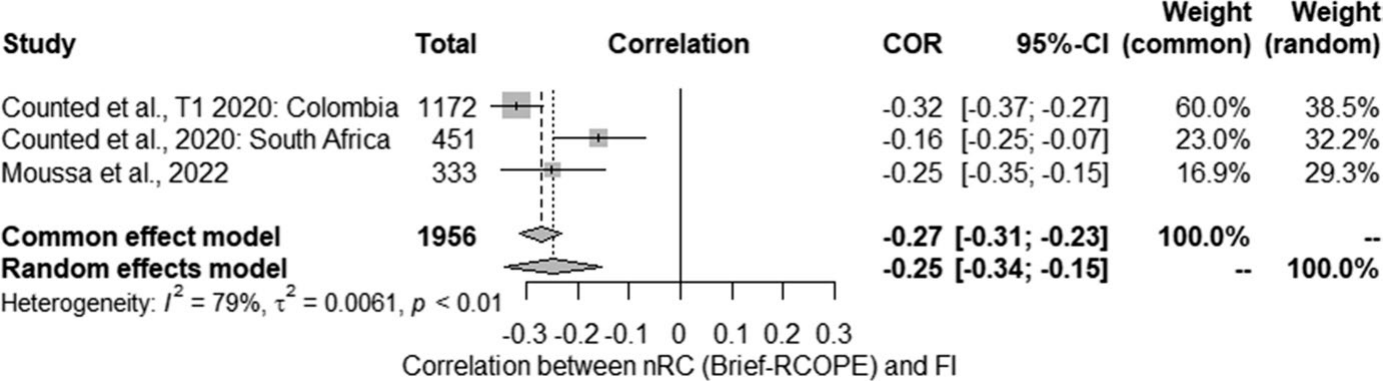
Negative religious coping and flourishing: forest plot

The meta-analysis of the relationship between level of flourishing and nRC (Brief-RCOPE) was conducted on 3 studies identified by the literature search as meeting the inclusion criteria. Their results were pooled to give a correlation of − 0.25 [− 0.34; − 0.15] suggesting a statistically significant negative relationship between these variables (*Z* = − 4.91; *p* < 0.001). (Fig. 4). Statistically significant heterogeneity was observed between studies (*Q* = 9.62; *p* < 0.05). The estimated amount of total heterogeneity was Tau^2^ = 0.006 and *I*^*2*^ = 79%.

Due to the heterogeneity of the results, potential moderators were also analysed in more detail: the percentage of women, relationship status (percentage of married people), and level of education (percentage of people with higher education). Unfortunately, due to deficiencies in the reported data, it was not possible to include more moderators. The tests for moderators showed that the percentage of women (*QM* (1) = 3.58; *p* = 0.058) and the percentage of married people (*QM* (1) = 3.21; *p* = 0.073) were at the statistical trend level. However, after taking into account the above moderators, residual heterogeneity decreased to an insignificant level: *Q* = 1.62 for percentage of women and *Q* = 1.73 for relationship status. In turn, higher education (*QM* (1) = 0.01; non-significant) was a statistically insignificant moderator.

**Fig. 5:**
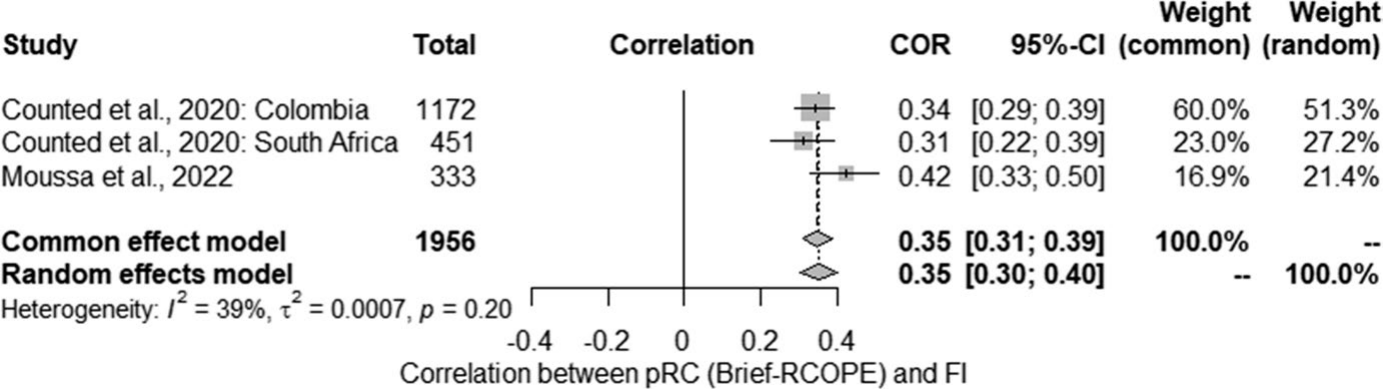
Relationship between flourishing and positive religious coping: forest plot

The next step was a meta-analysis of the relationship between pRC and levels of flourishing. The meta-analysis conducted for the relationship between flourishing and pRC (Brief-RCOPE) also included 3 studies. Studies identified in the literature search as meeting the inclusion criteria were pooled to give an overall correlation of 0.35 [0.30; 0.40] suggesting a statistically significant positive relationship (*Z* = − 3.18; *p* < 0.01). (Fig. 5). Statistically significant heterogeneity was not observed between studies (*Q* = 3.27; *p* > 0.05). The estimated amount of total heterogeneity was Tau^2^ = 0.0007 and *I*^2^ = 38.8%.

### Satisfaction with Life and Happiness

In the next step, the relationships of RC with SWL and happiness were analysed. The literature review identified 2 studies related to the relationship between RC and SWL and 1 related to the relationship between RC and happiness. The total number of participants in these 3 studies was 1789, and the research was conducted from April 2020 to March 2021. The Brief-RCOPE was used to evaluate RC in 2 of 3 studies, while happiness and SWL were analysed with different methods, which unfortunately made it impossible to carry out a meta-analysis of the collected data. All studies found relationships between RC and SWL and happiness. The exact results are shown in Table 3.

**Table 3:** Happiness, satisfaction with life, and religious coping

Authors [Country]	Sample *N* [group]	Date started*	Date finished*	Basic sociodemographic characteristics	Tools used	Main findings
Albani et al. (2022) [Greece]	200 [nursing students]	1 March, 2021	30 March, 2021	86.5% of the sample were female; mean age was 22.8 (*SD* = 12.2)	RC: Brief-RCOPE; subjective happiness scale	nRC was associated negatively with happiness
Dobrakowski et al. (2021) [Poland]	365 [Catholics]	1 April, 2020	30 September, 2020	75% of the sample were women; mean age was 35.64 (*SD* = 14.55)	RC: Brief-RCOPE; SWL: BMLSS	pRC was positively correlated with life satisfaction, while nRC was negatively associated with SWL
Lopes and Nihei (2021) [Brazil]	1224 [undergraduate students]	14 September, 2020	19 October, 2020	68.6% of the sample were female; age: 18–24: 77.9%; > 24: 22.1%	RC: Brief-COPE; SWL: SWLS	RC was correlated positively with life satisfaction

*RC* religious coping, *Brief-RCOPE Brief* Religious COPE, *nRC* negative religious coping, *SWL* satisfaction with life, *BMLSS* The Brief Multidimensional Life Satisfaction Scale, *SWLS* satisfaction with life scale

*In studies that did not specify the exact dates on which data were collected (months only), the beginning (1) and end (30) of the month were used as the starting and ending points

### Post-traumatic Growth

The relationship between PTG and RC was analysed in 5 studies, in which 22,165 participants took part. The research was conducted from April 2020 to May 2021. The Brief-COPE was used to evaluate RC in all studies, while PTG was assessed using the Post-Traumatic Growth Inventory and Post-Traumatic Growth Index. Each of the studies showed a positive relationship between RC and PTG. The exact results are shown in Table 4. Unfortunately, due to the lack of data on the correlation coefficients, it was not possible to perform a meta-analysis.

**Table 4:** Post-traumatic Growth and RC

Authors [Country]	Sample *N* [group]	Date started*	Date finished*	Basic sociodemographic characteristics	Tools used	Main findings
Gupta et al. (2022) [whole world]	369 [hospitality and tourism lecturers]	1 February, 2021	30 April, 2021	53.66% of the sample were female; age 18–25: 16.53%; 26–35: 23.31%; 36–45: 27.9%; 46+ : 32.25%	RC: Brief-COPE; PTG: PTGI	RC was positively correlated with Post-Traumatic Growth Index
MacIntyre et al. (2020) [International]	634 [language teachers]	5 April, 2020	19 April, 2020	80% of the sample were female; age: < 22: 4%; 22–32: 19%; 33–43: 31%; 44–54: 32%; 55–65: 13%; > 65: 1%	RC: Brief-COPE; PTG: PTGI	RC was positively correlated with PTGI
Menculini et al. (2021) [Italy]	20,720 [GPs]	n.i	n.i	71% of the sample were female; mean age was 40.4 (*SD* = 14.3)	RC: Brief-COPE; PTG: PTGI-SF	RC was positively related to PTG: relating to others, new possibilities, personal strength, spiritual, and appreciation for life
Willey et al. (2022) [USA]	176 [older adults]	23 March, 2021	13 May, 2021	58% of the sample were female; mean age was 76.3 (*SD* = 8.94)	RC: Brief-COPE; PTG: PTGI;	RC was positively related to PTG
Yeung et al. (2022) [Hong Kong]	266 [Filipina domestic helpers]	9 May, 2020	17 May, 2020	n.i. about how many percent of the sample were female; age: 18–25: 0.8%; 26–35: 36.1%; 36–45: 41.3%; 46–55: 17.3%; > 55: 3.7%	RC: Brief-COPE; PTG: PTGI-SF	RC was positively related to PTG

*RC* religious coping, *PTG* post-traumatic growth, *n.i.* no information, *PTGI-SF* The Post-traumatic Growth Inventory-Short Form

*In studies that did not specify the exact dates on which data were collected (months only), the beginning (1) and end (30) of the month were used as the starting and ending points

## Discussion

The first part of this systemic review focused on research on the correlation between religious coping (RC) and positive mental health (MH) indicators. The analysis of the literature allowed us to identify the following studies that met the inclusion criteria: 5 studies on the relationship between RC and quality of life (QoL), 10 studies on the relationship between RC and well-being (WB), 2 studies related to the relationship between RC and satisfaction with life, 1 related to the relationship between RC and happiness, and 5 studies on the relationship between RC and post-traumatic growth (PTG). It was only possible to conduct a meta-analysis on the relationship between negative religious coping (nRC) and flourishing, which found a value of *r* = − 0.25 [− 0.34; − 0.15]; however, the results were heterogeneous. The percentage of women in the studies and the percentage of married people used in the analysis as moderators were on the verge of statistical tendency. On the other hand, for the relationship between positive religious coping (pRC) and flourishing, the results were homogeneous and indicated a relationship of *r* = 0.35 [0.30; 0.40].

The synthesis of data from studies covering the relationship between RC and QoL indicated that the vast majority of studies conducted in this area were cross-sectional. Only the study by Schamblaw et al. (2021) had a follow-up after 1 month. Data from cross-sectional studies were collected from different populations (including students, people with chronic illnesses, and GPs) in different countries and with the use of different questionnaires; in most cases, these were tools assessing pRC/nRC, and two used the Brief-COPE. It should be noted that in most of the cross-sectional studies, a relationship was observed between QoL and RC, while the data from the longitudinal study did not find a relationship between RC and QoL. Data from a longitudinal study by Danhauer et al. (2009) on women with breast cancer indicate that coping strategies predict QoL to a limited extent, while QoL may predict coping strategies equally or to a greater extent. In turn, the studies by Pargament et al. (2004) and Trevino et al. (2010) indicate a weak association between QoL and pRC/nRC. It should be noted that the coping process is dynamic, and both the strategies used and the extent to which they are used may change over time depending on the effects achieved. This is important not only for the analysis of possible relationships or the impact of a given strategy, but also due to the content of the items used in the questionnaires to assess pRC/nRC. The content of items in these questionnaires may measure the effects of coping with a given stressor to a greater extent than the strategy used; for example "Felt punished by God for my lack of devotion" or "Wondered what I did for God to punish me" (Brief-RCOPE) indicate a failure to cope with a given stressor—that is, they seem to reflect the transaction effect (Lazarus & Folkman, 1984) to a greater extent than the methods used to reduce the level of stress or regulate emotions. At the same time, it should be noted that the analysis did indicate such a relationship, but the data from the longitudinal study may indicate that the choice of this strategy was more dependent on QoL; however, this requires further additional investigation due to limited data.

Studies assessing the relationship between RC and WB were also conducted around the world in different populations throughout the entire period of the pandemic. Most of these studies were cross-sectional, except for one longitudinal study (Davis et al., 2021). The cross-sectional studies mostly indicated the presence of this relationship, while longitudinal studies indicated no such relationship. As in the case of QoL, we should wonder to what extent the degree of RC use may be determined by the current state of the participant. The collected data also allowed for a meta-analysis of the results of 3 studies on the relationship between flourishing and p/nRC. However, these results should be approached with caution due to the limited data included in the analysis. In the case of nRC, statistically significant heterogeneity was observed, which could not be explained with the use of moderators: the percentage of women and married people reached the level of statistical trend, while the level of education was statistically insignificant. It should be noted that a very limited number of variables were used as moderators due to uncontrolled data or deficiencies in reporting data describing the studied group, which could be further analysed as a potential source of variability in the research results. However, all three studies showed a negative relationship. A meta-analysis of studies assessing the relationship between pRC and flourishing showed a weak positive relationship, with the results being homogeneous. In conclusion, the collected data indicate the existence of a relationship between WB and RC, and the nature of this relationship requires more analysis—longitudinal data do not indicate that RC determines changes in WB. Meta-analyses confirm a positive relationship between pRC and flourishing irrespective of sociodemographic factors, and the strength of the correlation between nRC and flourishing seems to depend on factors that could not be determined due to gaps in the reported data.

Next, satisfaction with life (SWL) and happiness were analysed. Cross-sectional studies covering this topic were conducted in various countries, most of them (*n* = 2) on the student population. The synthesis of the results allows us to draw conclusions about the relationship between these constructs: both neutral RC and pRC were positively associated with SWL, while nRC was negatively associated with levels of happiness and SWL. It should be noted that different assessment tools were used to assess both SWL and RC. The lack of longitudinal studies prevents conclusions being drawn about the direction of this relationship and its possible impact.

The last of the positive MH indicators included in this review was post-traumatic growth (PTG). The review of the studies allowed us to identify 5 cross-sectional studies conducted in different countries on very diverse populations. All studies assessed RC using the Brief-COPE, but meta-analysis could not be performed due to deficiencies in the reported correlation coefficients. All studies clearly indicated a positive relationship between PTG and RC. Unfortunately, it was not possible to identify longitudinal studies that would allow the assessment of the impact or direction of this relationship. Earlier longitudinal studies performed in clinical trials indicated no relationship (Scrignaro et al., 2011) or a negative relationship between RC and PTG (Rzeszutek et al., 2017). Studies conducted on a population of natural disaster survivors indicate the beneficial effects of pRC (Arkin, 2022; Chan et al., 2013). It would be valuable to further explore this relationship: whether it depends on an objective/subjective assessment of a given event, the nature of its causes, or whether such differences may result from the methodology used. It should be noted that in the case of the first two examples, the Brief-COPE was used, in which the RC items are formulated in a neutral way, and in the case of hurricane victims, RC was assessed in terms of p/nRC.

To sum up, the coping process usually involves a whole range of different types of strategies based on, inter alia, what resources are available (e.g. social support), how they can be used, as well as the expected effectiveness of their use. For almost all people, the pandemic was a novel situation where previous strategies did not necessarily have the expected results, which could have been an additional source of stress. The vast majority of studies included in the review suggested either no or very weak relationships between positive MH indicators and RC. The exception seems to be PTG, for which cross-sectional studies showed a beneficial effect. Single longitudinal studies indicated no influence of RC on the analysed dependent variables (QoL, WB), but these results should certainly be replicated. It is also worth noting that most analyses were based on the variable-centred approach, focusing on the percentage of variation in dependent variables that was explained by coping strategies; but it is worth remembering that they occur in particular combinations. It would also be worth considering the analysis of subgroups that differ in their degree of use of specific strategies with the use of methods allowing for the identification of such profiles (k-means clustering; LPA, LCA).

### Study Limitations

The cross-sectional nature of most of the analysed studies prevents inferences about the direction of the relationship between the variables included in the review. The use of this strategy results primarily from the highly burdensome nature of the stressor, which may be largely related to the observation of artificial positive correlations between RC and worse mental health indicators in cross-sectional studies. These types of results may also lead to overlooking the positive aspects that RC may have on mental health in the long term.

The most important limitation of this review is the small number of studies (*n* = 3) included in the calculations; the results of the meta-analyses should therefore be interpreted with caution. Another serious limitation is the heterogeneity obtained in one of the meta-analyses, which could not be explained based on the available moderators. Another factor influencing the quality of the obtained results is directly related to this: unfortunately, in many cases, the basic data describing the sample were not reported, so these variables could not be used as moderators in meta-regression. Correlation matrices between the analysed variables were not reported in many studies, which limited the possibilities for further data analysis. Authors should keep in mind that sample description and data reporting should allow for both replication and further analyses: the results obtained in a given study may depend on many factors related to, for example, the selection of the sample or the time and place they were carried out; only a further collective analysis of the results of many studies will allow us to draw more precise conclusions.

## Conclusions

The collected data suggest that there is a relationship between RC and positive MH indicators. Data from longitudinal studies in turn suggest that RC is not associated with levels of positive MH indicators. Meta-analyses indicate that the relationship between pRC and flourishing is less sensitive to differences in sample structure, and the association between nRC and flourishing may be partially dependent on some sociodemographic indicators; however, this requires further analysis. Due to the shortcomings in reporting both analyses and data on the sample structure, the results should be interpreted with caution.

## Figures and Tables

**Fig. 6:**
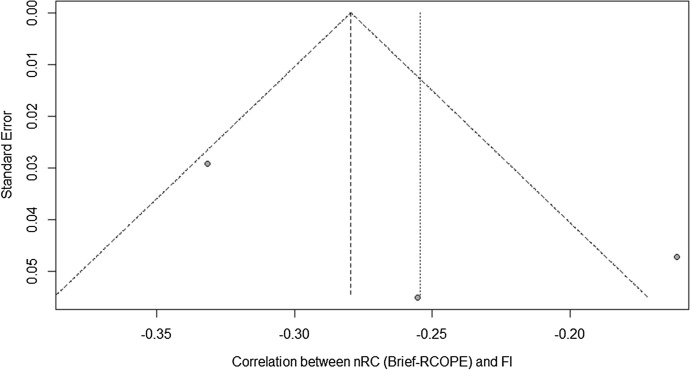
Negative religious coping and flourishing: funnel plot

**Fig. 7:**
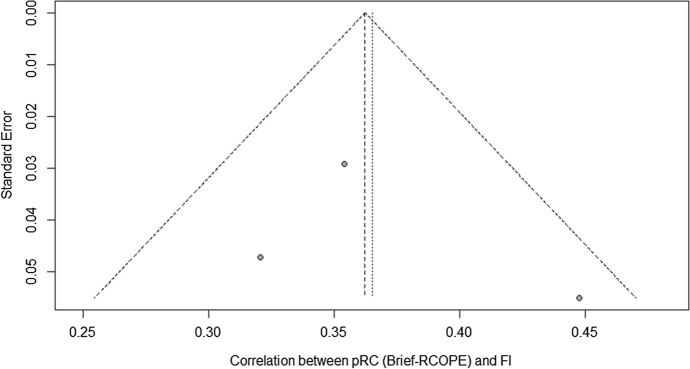
Relationship between flourishing and positive religious coping: forest plot

**Fig. 8:**
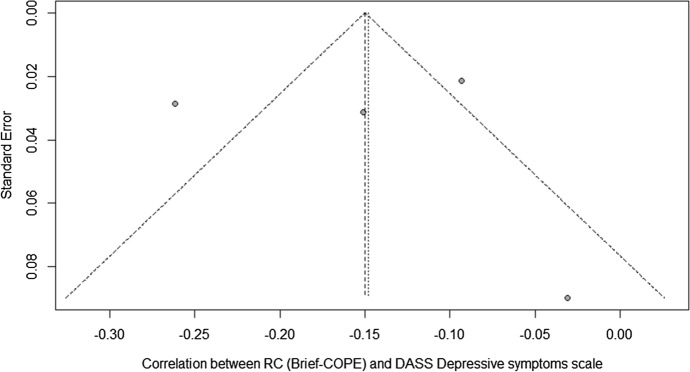
Religious coping and severity of depressive symptoms: funnel plot

**Fig. 9:**
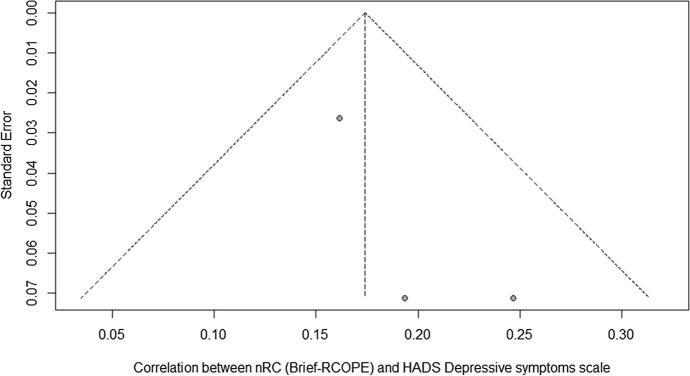
Relationship between negative religious coping and severity of depressive symptoms: funnel plot

**Fig. 10:**
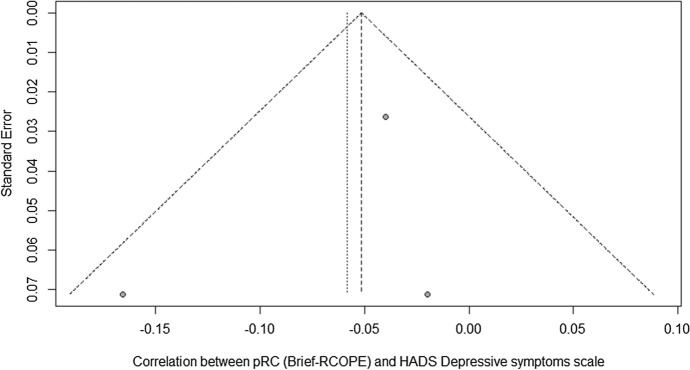
Relationship between positive religious coping and severity of depressive symptoms: funnel plot

**Fig. 11:**
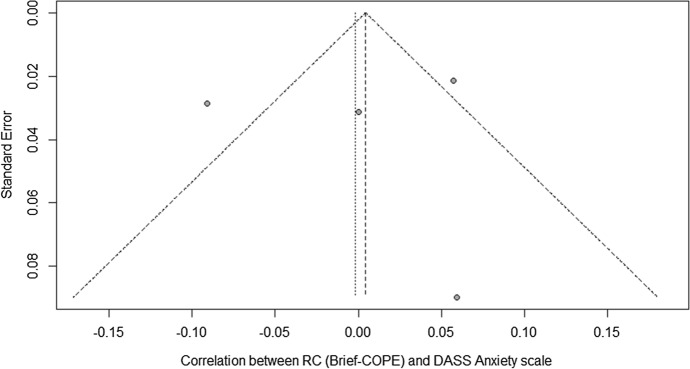
Religious coping assessed with Brief-COPE and anxiety: funnel plot

**Fig. 12:**
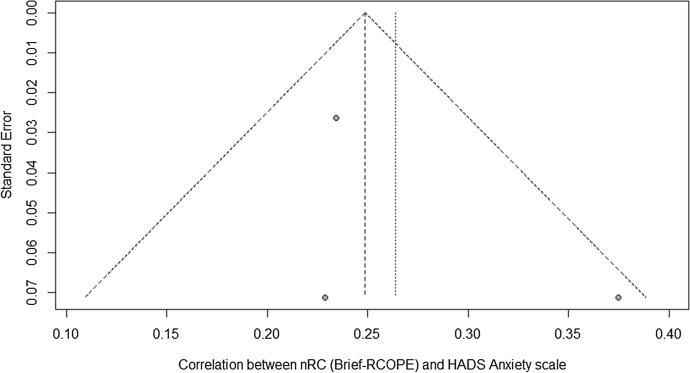
Negative religious coping and anxiety: forest plot

**Fig. 13:**
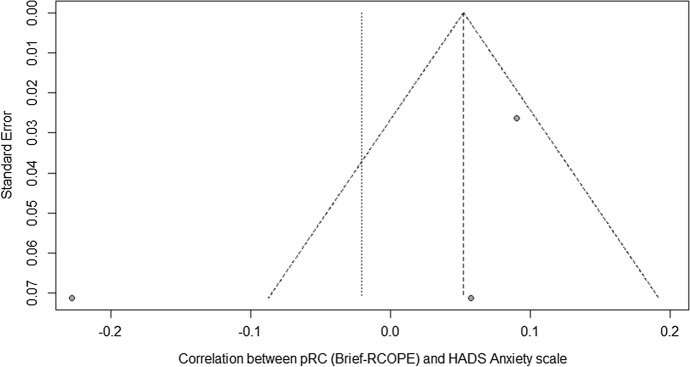
Positive religious coping and anxiety: forest plot

**Fig. 14:**
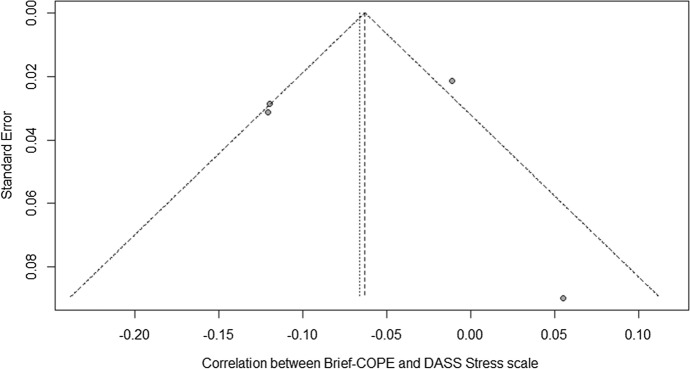
Religious coping and level of stress: forest plot

## Data Availability

Data supporting findings are available at: osf.io/54ygr (https://doi.org/10.17605/OSF.IO/GMNFV).

## Appendices

### Appendix 1

**Table:**

Authors [Country]	Selection				Comparability	Outcome		Sum
	1	2	3	4	1	1	2	
Albani et al. (2022)	1	0	0	2	1	1	1	6
Alsolais et al. (2021)	1	1	0	2	1	1	1	7
Altunan et al. (2021)	1	1	0	2	1	1	1	7
Anjum et al. (2022)	1	0	0	2	1	1	0	5
Awoke et al. (2021)	1	1	0	2	2	1	1	8
Babore et al. (2020)	1	1	0	2	2	1	1	8
Bakır et al. (2021)	1	1	0	2	1	1	1	7
Besirli et al. (2021)	1	0	0	2	1	1	1	6
Bianchi et al. (2021)	1	1	0	2	2	1	1	8
Budimir et al. (2021)	1	1	0	2	1	1	1	7
Cansız et al. (2021)	1	1	0	2	1	1	1	7
Captari et al. (2022)	1	0	0	2	2	1	1	7
Chow et al. (2021)	1	0	0	2	2	1	1	7
Chui et al. (2021)	1	1	0	2	2	1	1	8
Counted et al. (2022)	1	0	0	2	2	1	1	7
Davis et al. (2021)	1	1	0	2	2	1	1	8
Dobrakowski et al. (2021)	1	0	0	2	2	1	1	7
Eisenbeck et al. (2022)	1	1	0	2	2	1	1	8
El Tahir et al. (2022)	1	0	0	2	1	1	1	6
Faronbi et al. (2021)	1	1	0	2	1	1	1	7
Fukase et al. (2022)	1	1	0	2	1	1	1	7
Ghoncheh et al. (2021)	1	1	0	2	2	1	1	8
Girma et al. (2021)	1	1	0	2	1	1	1	7
Gupta et al. (2022)	1	0	0	2	1	1	1	6
Habib et al. (2020)	1	0	0	2	1	1	1	6
Jarego et al. (2021)	1	1	0	2	2	1	1	8
Kandeğeret al. (2021)	1	0	0	2	1	1	1	6
Lopes et al. (2021)	1	1	0	2	2	1	1	8
MacIntyre et al. (2020)	1	0	0	2	2	1	1	7
Mahamid et al. (2021)	1	0	0	2	1	1	1	6
Margetić et al. (2022)	1	1	0	2	2	1	1	8
Masha'al et al. (2022)	1	0	0	2	1	1	1	6
Menculini et al. (2021)	1	1	0	2	1	1	1	7
Mestas et al. (2021)	1	0	0	2	2	1	1	7
Mishra et al. (2021)	1	1	0	2	2	1	1	8
Moussaet al. (2022)	1	1	0	2	1	1	1	7
Narendra et al. (2022)	1	1	0	2	2	1	1	8
Park et al. (2021)	1	1	0	2	2	1	1	8
Penengo et al. (2021)	1	1	0	2	2	1	1	8
Quansah et al. (2022)	1	0	0	2	2	1	1	7
Rahimi et al. (2021)	1	1	0	2	2	1	1	8
Romdhane and Cheour (2021)	1	0	0	2	2	1	1	7
Romero-García et al. (2022)	1	1	0	2	1	1	1	7
Rosa-Alcázaret al. (2021)	1	0	0	2	2	1	1	7
Shamblaw et al. (2021)	1	1	0	2	2	1	1	8
Shehata et al. (2021)	1	0	0	2	1	1	1	6
Sitarz et al. (2021)	1	0	0	2	1	1	1	6
Smida et al. (2021)	1	0	0	2	1	1	1	6
Thomas et al. (2020)	1	0	0	2	1	1	1	6
Umucuet al. (2020)	1	0	0	2	1	1	1	6
Vancappel et al. (2021)	1	0	0	2	2	1	1	7
Vannini et al. (2021)	1	0	0	2	2	1	1	7
Vitorino et al. (2021)	1	0	0	2	2	1	1	7
Willey et al. (2022)	1	0	1	2	2	1	1	8
Williams et al. (2021)	1	0	0	2	2	1	1	7
Yee et al. (2021)	1	1	0	2	2	1	1	8
Yeung et al. (2022)	1	1	0	2	2	1	1	8
Yıldırım et al. (2021)	1	0	0	2	2	1	1	7
Zarrouq et al. (2021)	1	1	0	2	1	1	1	7

### Appendix 2: Funnel Plots of Analysed Studies

The results should be interpreted with caution due to the small number of studies.

See Figs. 6, 7, 8, 9, 10, 11, 12, 13, 14.

### Appendix 3: Number of STUDIES and participants Conducted per Country

**Table:**

	Number of studies	Participants
Algeria	1	253
Argentina	1	145
Australia	2	204
Austria	1	1005
Bangladesh	1	344
Brazil	3	2678
Canada	2	1129
Colombia	3	2456
Croatia	1	2860
Egypt	2	568
Ethiopia	2	950
France	2	1475
Germany	1	281
Ghana	1	760
Greece	1	200
Hong Kong	1	266
Hungary	1	262
India	2	1184
Indonesia	1	277
Iran	1	696
Italy	5	24,071
Japan	1	1468
Jordan	1	282
Lebanon	2	627
Malaysia	5	2210
Mexico	2	1395
Morocco	1	1435
New Zealand	1	43
Nigeria	2	707
Pakistan	3	940
Palestine	1	400
Poland	3	2812
Portugal	2	913
Qatar	2	227
Romania	1	546
Russia	1	307
Saudi Arabia	2	592
Slovenia	1	1271
South Africa	2	902
Spain	3	1196
Sweden	1	278
Thailand	1	405
Tunesia	1	603
Turkey	7	2427
UK	1	382
United Arab Emirates	1	543
USA	6	2918

## Declarations

### Clinical trial registration

This Review was pre-registered at OSF: osf.io/54ygr (https://doi.org/10.17605/OSF.IO/GMNFV).
